# Functional improvement by body-powered 3D-printed prosthesis in patients with finger amputation: Two case reports

**DOI:** 10.1097/MD.0000000000029182

**Published:** 2022-06-24

**Authors:** Min-Yong Lee, Seung Hak Lee, Ja-Ho Leigh, Hyung Seok Nam, Eun Young Hwang, Jung Yeon Lee, Sol Han, Gangpyo Lee

**Affiliations:** a Department of Rehabilitation Medicine, Seoul National University Hospital, Seoul, Korea; b Rehabilitation Center, Incheon Workers’ Compensation Hospital, Incheon, Korea; c Department of Rehabilitation Medicine, Asan Medical Center, Seoul, Korea; d Department of Rehabilitation Medicine, National Traffic Injury Rehabilitation Hospital, Yangpyeong, Korea; e Department of Rehabilitation Medicine, UAE Sheikh Khalifa Specialty Hospital, RAK City, UAE; f Rehabilitation Medicine Research Center, Incheon Workers’ Compensation Hospital, Incheon, Korea.

**Keywords:** 3D printing, amputation, finger injury, prosthesis

## Abstract

**Rationale:**

The most common upper limb amputations are finger amputations, resulting in functional limitations that lead to problems with activities of daily living or job loss. For many years, prosthetic options for finger amputations have been limited to passive prostheses. In many countries including South Korea, body-powered finger prostheses have rarely been prescribed due to high cost, lack of experience of physicians and prosthetists, low interest and no coverage by insurance benefits. We report 2 cases of work-related finger amputations in patients who received body-powered 3D-printed finger prostheses.

**Patient concerns and diagnosis:**

Patient 1 was a 25-year-old woman with second and third finger amputations at the proximal interphalangeal level. Patient 2 was a 26-year-old man who sustained a second finger amputation at proximal interphalangeal level.

**Interventions:**

We created body-powered 3D-printed finger prostheses that mimicked distal interphalangeal joint motion through patient-driven metacarpophalangeal joint motion using a string connected to a wrist strap and a linkage system. The source code “Knick Finger” was downloaded from e-NABLE.

**Outcomes:**

After 1 month of prosthesis training, both patients were satisfied with the prostheses and showed improved performance in patient-derived goals of cooking (patient 1) and typing on a computer (patient 2).

**Lessons:**

Over the past decade, significant advances have been made in 3D-printed prosthetics owing to their light weight, low cost, on-site fabrication, and easy customization. Although there are still several limitations in the general application of 3D-printed finger prostheses, our study suggests that for patients with finger amputations, body-powered 3D-printed finger prostheses have high potential as an additional prosthetic option to the existing passive cosmetic prostheses.

## 1 Introduction

Finger amputations are the most common upper limb amputations and usually occur because of traumatic injuries.^[1]^ Most finger amputations are work related. In South Korea, approximately 5000 work-related finger amputations occur each year, accounting for 93.7% of the total work-related limb amputations.^[2]^ Even though injured hands with finger amputations may be functional when more than half of the proximal phalanx is preserved,^[3]^ loss of even a small part of 1 finger can result in functional limitations and lead to problems with activities of daily living or job loss.^[4]^

While the incidence of finger amputations is much higher than that of arm amputations, the development of options for finger prostheses is relatively insufficient. This is due to several factors, including suspension difficulty, loss of proprioception, aesthetics, and discomfort. Therefore, passive prostheses (mainly silicone cosmetic prostheses) have been the only option for a long time despite limited functional support.^[5]^ In a few cases, body-powered prostheses [eg, the M-Finger (Partial Hand Solutions LLC), the Naked Finger (Naked Prosthetics Incorporated)], or externally powered prostheses have been used for functional improvement. However, in many countries including South Korea, body-powered prostheses have rarely been prescribed for several reasons, including high cost, no coverage by insurance benefits, and lack of experience of physicians and prosthetists.

Over the past decade, significant advances have been made in 3D-printed prosthetics owing to their light weight, low cost, on-site fabrication, and easy customization.^[3,6]^ The e-NABLE, a global community that creates and shares open-source designs for assistive devices, has promoted the development of 3D-printed prostheses.^[7]^ Various designs of 3D-printed prosthetic finger have been published. However, in clinical practice, few cases of applying these prostheses and evaluating the effectiveness have rarely been reported.

In this study, we report 2 cases of work-related finger amputation in patients who received body-powered 3D-printed finger prostheses and underwent sufficient prosthetic training. We fabricated 3D-printed finger prostheses using a source code downloaded from e-NABLE.

## 2 Materials and methods

### 2.1 Body-powered 3D-printed finger prosthesis

**Figure 1:**
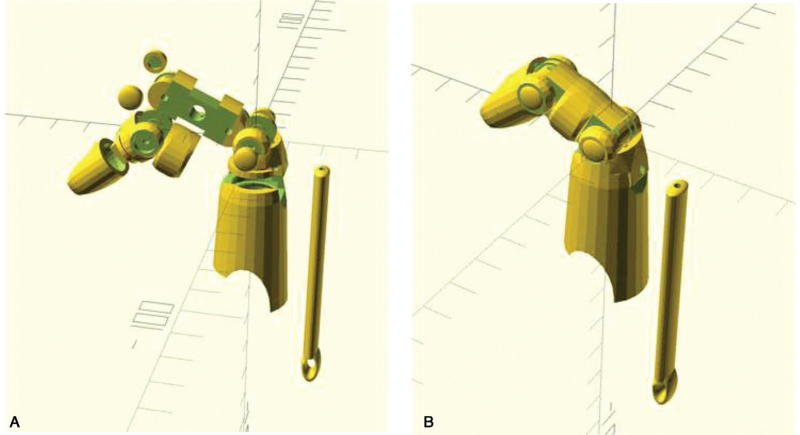
Modeling of the body-powered 3D-printed prosthetic finger using OpenSCAD. (A) Individualized prosthetic parts. (B) Assembly of individualized prosthetic parts.

We created body-powered 3D-printed finger prostheses that mimicked distal interphalangeal joint motion through patient-driven metacarpophalangeal (MCP) joint motion using a string connected to a wrist strap and a linkage system. The stump sizes of the fingers were measured for parametric modeling using 9 parameters (socket width, depth, middle section length, etc). Parametric modeling was performed using the free 3D CAD software, OpenSCAD,^[8]^ and individualized prosthetic parts were fabricated using a fused filament fabrication type 3D printer (Cubicon, Single plus, Korea) (Fig. 1). The source code “Knick Finger”, was downloaded from e-NABLE.^[9]^ Acrylonitrile butadiene styrene resin was used for the hard portion and thermoplastic polyurethane resin was used for the soft portion. Fishing line and rubber strings were used for assembly. The time from measurement to prosthesis production was 1 day. The total cost was approximately $30 per 3D-printed finger prosthesis.

### 2.2 Clinical assessment

To evaluate the clinical improvement, stump pain, range of motion (ROM), hand function, and occupational performance were assessed. Stump pain was assessed using the Visual Analog Scale which ranges from 0 (no pain) to 10 (extreme pain).^[10]^ Hand function was assessed using the Box and Block Test and the Jebsen–Taylor Hand Function Test (JHFT). The Box and Block Test, which measures unilateral gross manual dexterity, requires the subject to move, one by one, the maximum number of blocks from 1 box to the adjacent box, within 60 seconds.^[11]^ The JHFT is an objective measure of fine and gross motor hand function using simulated activities of daily living (7 subsets) and is performed on both hands. The total JHFT score is the sum of the time to complete each of the 7 subsets.^[12]^ Occupational performance was assessed using the Canadian occupational performance measure, an evidence-based outcome measure that assesses a subject's self-perception of performance and satisfaction in everyday living.^[13]^

## 3 Case presentation

We report 2 partial hand amputees with prosthetic fingers. The local institutional review board approved this study (no. 1902-094-1009) Informed consent for the publication of clinical data was obtained from each patient.

**Figure 2:**
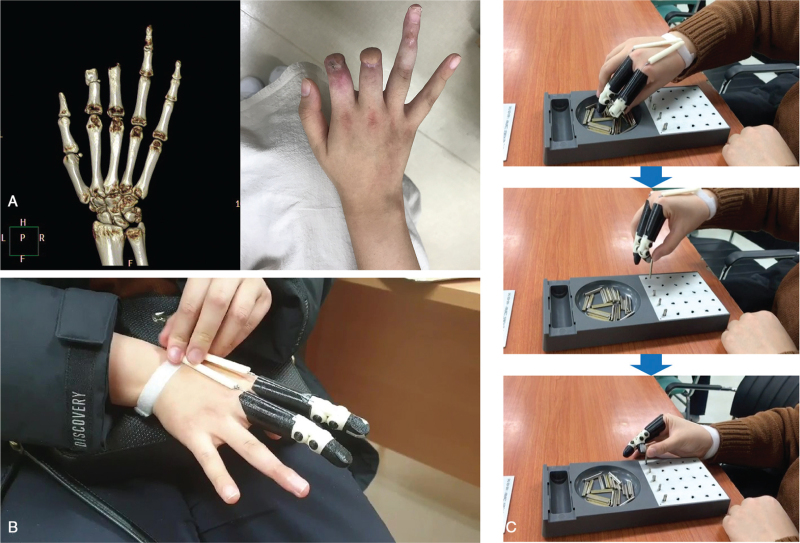
The case of patient 1. (A) A 3D-reconstructed bone structure from a computed tomographic scan and gross appearance of the right hand in patient 2. The amputation levels correspond to second and third proximal phalanges. (B) The patient is wearing the fabricated body-powered 3D-printed finger prostheses. (C) The patient is performing peg board training with the finger prostheses.

Patient 1 was a 25-year-old woman who sustained a blender injury to the right (dominant) hand at work, resulting in amputation of the second, third, and fourth fingers. On the same day as the injury, the fourth finger was replanted, but the second and third fingers were amputated at the proximal interphalangeal (PIP) level (Fig. 2A). After 6 months, she was transferred to our rehabilitation center with stump pain and limited ROM in the second and third MCP joints. The patient wanted the focus of rehabilitation to be on writing, typing, and cooking. Intensive hand rehabilitation was initiated, including aggressive passive and active ROM exercises of the fingers. After 2 months of intensive hand rehabilitation, she showed significant improvement in stump pain, ROM, hand function, and occupational performance (Table 1). Since all finger ROM was restored, we fabricated 3D-printed prostheses for the second and third fingers. The patient received additional rehabilitation with prosthesis training for 1 month (Fig. 2B and C) (Video S1, Supplemental Digital Content). After training, the occupational performance of cooking showed additional improvements, and the patient was very satisfied. However, there was no definite improvement in the JHFT (Table 1).

**Table 1:** Clinical evaluation of patient 1.

	1st visit		Preprosthetic (2 mo after 1st visit)		Postprosthetic (3 mo after 1st visit)	
Stump pain (VAS)	7		0		0	
ROM (°)						
2nd MCP	55		80		80	
3rd MCP	60		85		90	
BBT	33		55		49	
JHFT (total score)	56.40		63.68		56.35	
COPM (score)	Performance	Satisfaction	Performance	Satisfaction	Performance	Satisfaction
Writing	2	1	10	9	10	10
Typing	5	3	8	8	8	8
Cooking	1	1	1	1	7	7

BBT = Box and Block Test, COPM = Canadian occupational performance measure, JHFT = Jebsen–Taylor hand function test, MCP = metacarpophalangeal, ROM = range of motion, VAS = visual analog scale.

**Figure 3:**
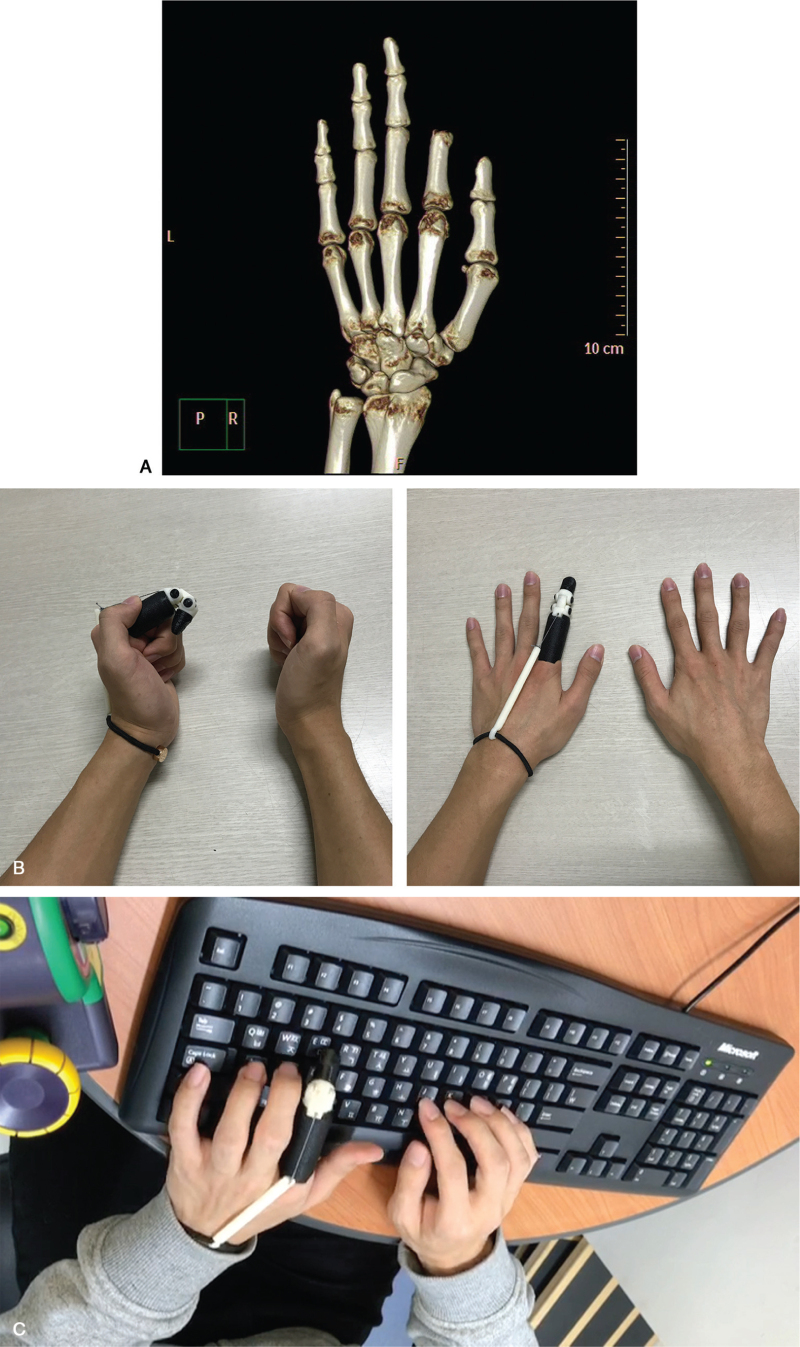
The case of patient 2. (A) A 3D-reconstructed bone structure from a computed tomographic scan of the left hand in patient 2. The amputation levels correspond to the second proximal phalanx. (B) The patient is wearing a body-powered 3D-printed finger prosthesis. (C) The patient is typing on an electronic keyboard.

Patient 2 was a 26-year-old man who sustained a left second finger amputation during plumbing work. He underwent replantation surgery; however, necrosis occurred, and the patient required PIP joint disarticulation (Fig. 3A). He was transferred to our rehabilitation center 2 months after the injury with stump pain and no ROM restriction in the second MCP joint. After rehabilitation, he still desired improvements in tasks such as typing, cooking, and playing a guitar. As there was no restriction in the ROM of the second MCP joint at the initial visit, we immediately started designing the prosthesis. After 2 weeks, a 3D-printed finger prosthesis was provided (Fig. 3B). One month after rehabilitation focusing on prosthesis training, the patient showed significant improvement in stump pain, JHFT score, and occupational typing performance (Table 2). The patient reported that the prosthesis was especially useful for typing on a computer (Fig. 3C; Video S2, Supplemental Digital Content).

**Table 2:** Clinical evaluation of patient 2.

	Preprosthetic		Postprosthetic (1 mo later)	
Stump pain (VAS)	3		0	
JHFT (total score)	105.51		65.13	
COPM (score)	Performance	Satisfaction	Performance	Satisfaction
Typing	3	3	8	8
Playing a guitar	1	1	1	1

COPM = Canadian occupational performance measure, JHFT = Jebsen–Taylor hand function test, VAS = visual analog scale.

## 4 Discussion

These case reports showed that body-powered 3D-printed finger prostheses are feasible. These prostheses offer several advantages in clinical application. First, modeling of the prosthesis can be performed easily by measuring only 9 parameters, and only 1 day was required for fabrication with a 3D-printer. Second, the cost of the 3D-printed finger prosthesis in this study was very low (approximately $30) compared to the cost of a commercial body-powered prosthetic finger, which can range from $4000 to $10,000.^[14]^ Additionally, the prostheses showed significant functional effectiveness.

Patient 1 showed improved performance and satisfaction while wearing the prostheses when cooking, and patient 2 showed similar results when typing. In the case of young amputees who have jobs associated with hand function, such as typing, this body-powered finger prosthesis can be of great help in adjusting to the job rather than the aesthetic type.

However, 3D-printed prosthetics have some disadvantages compared to traditional prosthetics, such as poor durability, limited amount of materials, and inaccuracy.^[7]^ These factors limit their widespread use. Nevertheless, 3D-printed prosthetics are in high demand because the 3D-printed prosthesis can be an alternative for those who cannot afford to purchase commercial prosthetics and can be used as a transitional prosthesis before using an expensive commercial prosthesis.

A recent case study compared a body-powered 3D-printed partial finger prosthesis and a commercially available body-powered finger prosthesis [MCP-Driver (Naked Prosthetics Incorporated)] in a patient with an amputation at the PIP joint of the left second finger.^[15]^ The results of the study showed that the body-powered 3D-printed partial finger prosthesis produced functional improvements similar to a commercially available body-powered finger prosthesis. These results are similar to our results and demonstrate the functional effectiveness of the 3D-printed finger prostheses.

However, there are several limitations of 3D-printed finger prostheses. First, the current scientific evidence is insufficient. A randomized controlled study with a large sample size should be performed to confirm the clinical effectiveness. In addition, since the clinical manifestations of finger amputations vary, further investigations should be performed to validate the efficacy of using 3D-printed prostheses for various finger amputations. Second, although 3D-printed prostheses are relatively inexpensive, they lack durability. We did not analyze durability in this study. Poor durability could render 3D-printed prostheses as not cost effective. Therefore, durability must be improved through technological advances, and the durability and cost-effectiveness of 3D-printed prostheses should be analyzed in the future.

## 5 Conclusion

In conclusion, we provided body-powered 3D-printed finger prostheses and sufficient prosthetic training to 2 partial hand amputee. They showed improved function and satisfaction with the 3D-printed finger prostheses. We hope to provide a functional prosthetic finger at an affordable price. In the future, a large study with a 3D-printed finger prosthesis should be performed to confirm the clinical effectiveness and cost-effectiveness.

## Acknowledgments

The authors thank patients for their participation in this study.

## Author contributions

**Conceptualization:** Seung Hak Lee, Ja-Ho Leigh, Gangpyo Lee.

**Data curation:** Min-Yong Lee, Sol Han.

**Methodology:** Seung Hak Lee, Hyung Seok Nam, Eun Young Hwang, Jung Yeon Lee, Gangpyo Lee.

**Writing – original draft:** Min-Yong Lee, Seung Hak Lee.

**Writing – review & editing:** Min-Yong Lee, Gangpyo Lee.

## Supplementary Material

Supplemental Digital Content

## Supplementary Material

Supplemental Digital Content
